# Soybean and Lupine Addition in Hen Nutrition—Influence on Egg Immunoreactivity

**DOI:** 10.3390/molecules26144319

**Published:** 2021-07-16

**Authors:** Aneta Tomczak, Michalina Misiak, Magdalena Zielińska-Dawidziak

**Affiliations:** Department of Food Biochemistry and Analysis, Faculty of Food Science and Nutrition, Poznań University of Life Sciences, ul. Wojska Polskiego 28, 62-623 Poznań, Poland; aneta.tomczak@up.poznan.pl (A.T.); michalina.kmiotek@gmail.com (M.M.)

**Keywords:** egg allergens, soy allergens, egg proteins composition, egg protein immunoreactivity

## Abstract

Modifying hen fodder is a common way of changing eggs composition today. However, there is no information on the effect of the source of protein in the fodder replacement on egg allergenicity. This research aimed to detect potential differences in the immunoreactivity and protein composition of eggs from hens fed with fodder containing legume. The aim of the first step of the study was to select the proper solvent for extracting allergenic proteins from hen eggs. Two of them (containing Tween 20 and Triton 100) were selected, based on protein profile and concentration analysis. Egg-white- and egg-yolk-proteins extracts prepared with them were checked for potential differences, using SDS-PAGE electrophoresis, and then the Western-blot method, using sera from children allergic to eggs and soy. Preliminary studies on the influence of fodder composition on the composition of egg proteins suggest that the addition of soy and lupine to fodder modifies the expression of egg proteins. The observed differences in the immunoreactivity of proteins contained in hen egg-white samples do not seem to be as significant as the appearance of protein with a molecular weight of ~13 kDa in the yolk of eggs obtained from soybean-fed hens. This protein may increase the immunoreactivity of eggs for children allergic solely to soy.

## 1. Introduction

Hen eggs play an important role in human nutrition all over the world, as a basic component of a wide variety of food products [1]. Eggs are considered to be the perfect food, complying with the trends of consuming natural and minimally processed products. Bird eggs are a source of basic nutrients for humans: valuable proteins, lipids, minerals and vitamins necessary for proper development [2]. FAO/WHO [3] recognizes egg proteins as an international protein standard for adults because of the valuable amino acid composition. Due to their health-promoting properties discovered in recent years, eggs have been recognized as materials of both functional and nutritional importance [4]. Many new functions that were not previously mentioned have been assigned to eggs components, such as antimicrobial, anticancer, immunomodulating or antihypertensive action [2].

Simultaneously, hen eggs are the world’s second most-common food among the top eight food allergens [5], especially for children. Data from 2013 indicate that egg allergy affects up to 4% of children [6]. There are currently six accepted by WHO/IUIS whole-egg allergen determinants [3]. Egg white causes allergic reactions more strongly and more often than yolk, which does not change the fact that the yolk also contains allergenic substances [7]. According to two databases, namely Allergen WHO/IUIS (International Union of Immunological Societes) and Allergome, 15 chicken-egg allergens have currently been discovered, six of which (Gal d 1–6) are officially recognized by researchers meeting the purity of the test but also by rigorous analyzes. Most scientists recognize only the first four allergens that are considered to be the main causes of allergies in children, and Gal d 5 and 6 are considered adult allergens [7]. The four major hen-egg allergens have already been extensively studied and described (Gal d 1–4) [8]. Humans allergic only to egg white usually do not tolerate Gal d 2 (ovalbumin) and Gal d 1 (ovomucoid) [8], whereas allergy to Gal d 3–5 (ovotransferrin, α-lysozyme and livetin) is most common inpatients allergic to both egg white and egg yolk [2,9]. Patients diagnosed with egg allergy mostly show cross allergy to proteins of other bird eggs, due to the high homology of protein fractions. Antibodies present in the serum of people allergic to hen egg proteins cross-reacted strongly with proteins of turkey meat as well [10].

The literature data show the effect of laying-hen fodder on egg composition. Research involving the introduction of appropriate components into the fodder for laying hens clearly showed a modification of the fatty acid composition in the egg. Enriching eggs by modifying hen fodder, e.g., with fat-soluble vitamins (A, D, E, and K), microelements (iodine, selenium), n-3 polyenic acids and even caffeine, DHA or vitamin B12 [11] is a popular topic today. However, there is a lack of information about the influence of feeding on egg allergenicity.

The influence of fodder composition on immunoreactivity has been investigated in the case of milk proteins [12]. Research has shown that the addition of soy or lupine as a protein source to fodder modifies the composition of milk proteins. The presence of legumes in the fodder increased the immunoreactivity of raw milk for children allergic solely to soy (not to milk) and, at the same time, for children allergic to milk.

Therefore, a question arises whether changing hen-fodder composition by adding soybeans or narrow-leaved lupine may change the protein and peptide profile and whether it may have an impact on the immunoreactivity of eggs. The study aimed to detect potential differences in the immunoreactivity, protein and peptide composition of eggs from hens fed with fodder containing legume seeds.

## 2. Results

### 2.1. Extraction and Protein Quantification

The first stage of the experiment presents the selection of a proper extractant of egg protein. Based on the literature data, six different extractants were applied: PBS—phosphate-buffered saline [13]; PBST—pH 7.4, i.e., PBS buffer with 0.05% of Tween 20 [14]; H_2_O—acidified with 1M HCl dH_2_O, pH 6 [15]; SDS—4% SDS [16]; TT—1% Tween 20 + 0.4% Triton x-100 [17]; and TN—1% Tween 20 + 0.4% Triton X-100, 280 mM NaCl, 40 mM NaH_2_PO_4_, pH 7.4 [18]. They were used for the extraction of both proteins from egg white and yolk. Eggs purchased in a commercial network were used for this preliminary experiment. The appropriate extractant was selected [14] based on three criteria: (1) the protein concentration in the extracts, (2) the amount of protein fractions and (3) the quality of their separation in a 14% polyacrylamide gel. Protein content dependence on the extractant used is summarized in Table 1.

**Figure 1:**
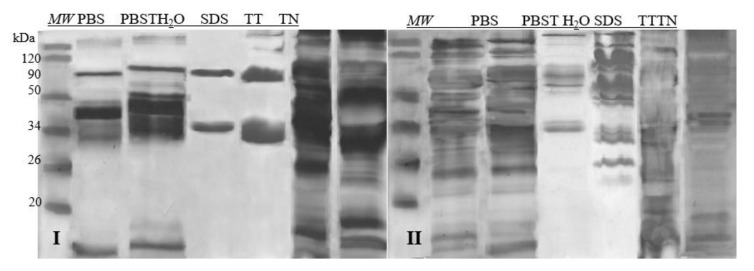
SDS-PAGE separations proteins profiles of samples prepared with6 different extractants. I—egg white, II—egg yolk, *MW*—molecular weight marker; PBS—phosphate-buffered saline; PBST—pH 7.4 (PBS buffer with 0.05% of Tween 20); H_2_O dd—acidified with 1M HCl dH_2_O, pH 6; SDS—4% SDS; TT—1% Tween 20 +0.4% Triton x-100; TN—1% Tween 20 + 0.4% Triton X-100, 280 mM NaCl, 40 mM NaH_2_PO_4_, pH 7.4.

Taking into account the number of fractions and the quality of their separation on the gel after the SDS-PAGE separation, the best extractant turned out to be TN and TT. Based on the gel analysis with the CLIQS software, the highest number of protein fractions were extracted after the application of those two extractants: 8 and 9 fractions in the case of egg white, and 12 and 15 in the case of egg yolk, respectively. The separation of proteins during electrophoresis was precise, which was convenient for further qualitative analyses (Figure 1). Therefore, these two solvents were used in further experiments.

### 2.2. SDS-PAGE Electrophoresis

#### 2.2.1. Egg-White Proteins

**Figure 2:**
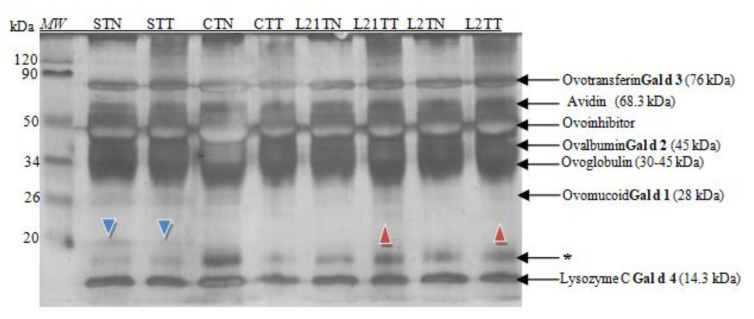
SDS-PAGE separation protein profiles for egg white after silver staining. Hen eggs were obtained after feeding the hens with soy, lupine or without adding legumes: S—soy for 21 days; C—control without legumes; L2—lupine for 2 days; L21—lupine for 21 days; TT—1% Tween 20 +0.4% Triton x-100; TN—1% Tween 20 + 0.4% Triton X-100, 280 mM NaCl, 40 mM NaH_2_PO_4_, pH 7.4, *MW*—molecular weight marker, * unknown protein fraction.

Afterwards, the extracts of the target samples were subjected to the SDS-PAGE analysis. Based on the molecular weight analysis and available data from the literature [3,19], it was possible to identify the main proteins in the egg white (Figure 2).

All of the known hen egg-white allergens were identified in the prepared samples (Figure 2). Moreover, the differences in the protein profile between C, S, L2 and L21 were noted. An interesting new protein fraction of ~20 kDa was indicated (blue arrowhead) in the samples of hen egg-white protein obtained from hens fed with soybean fodder. 

A difference was observed also in the case of hen egg-white samples, where there was an addition of lupine into fodder: L21TT and L2TT. Small amounts of a different protein fraction with amass of ~24 kDa were identified in them as well (red arrowhead). Simultaneously, a decrease in the content of the protein with the molecular mass ~17 kDa (marked by an asterisk) compared to their content in control eggs was observed, especially in eggs obtained from hens fed with soybean-supplemented fodder.

**Figure 3:**
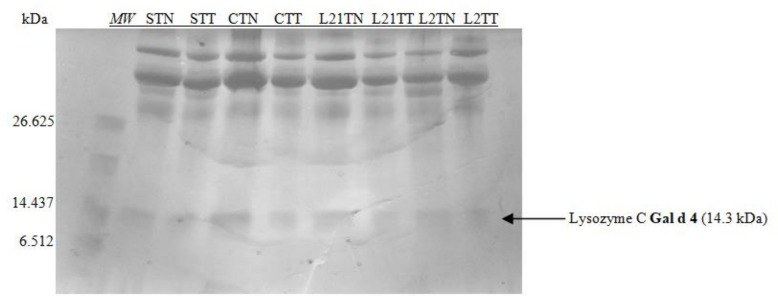
SDS-PAGE separation peptide profiles with Coomassie staining for egg white. Hen eggs were obtained after feeding the hens with soy, lupine or without adding legumes: S—soy for 21 days; C—control without legumes; L2—lupine for 2 days; L21—lupine for 21 days; TT—1% Tween 20 + 0.4% Triton x-100; TN—1% Tween 20 + 0.4% Triton X-100, 280 mM NaCl, 40 mM NaH_2_PO_4_, pH 7.4, *MW*—molecular weight marker.

The obtained extracts were also subjected to electrophoresis in 16.5% MP Tris-Tricine gel dedicated for the separation of peptide fractions in the range of 1.4–26.6 kDa (Figure 3).

Considering the size marker, only one allergenic protein was identified on the gel, which indicates the separation of lysozyme with the molecular weight of ~14 kDa. The differences in the protein profile after this kind of electrophoretic separation are invisible. This can be explained by the method of staining (the sensitivity of the dye), or by the structure of the gel—the accumulation of proteins in the gel’s upper part.

#### 2.2.2. Egg Yolk Proteins

**Figure 4:**
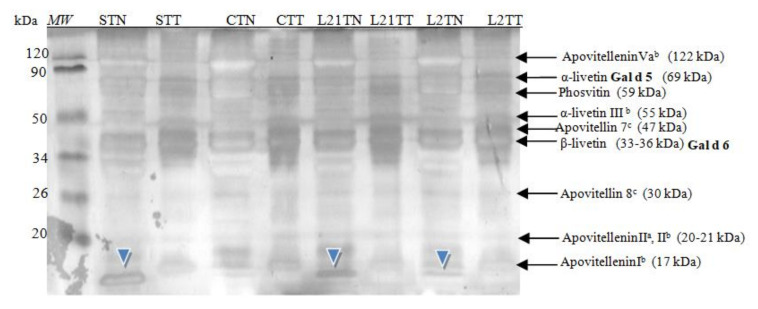
SDS-PAGE separation protein profiles for egg yolk after silver staining. Hen eggs were obtained after feeding the hens with soy, lupine or without adding legumes: S—soy for 21 days; C—control without legumes; L2—lupine for 2 days; L21—lupine for 21 days; TT—1% Tween 20 + 0.4% Triton x-100; TN—1% Tween 20 + 0.4% Triton X-100, 280 mM NaCl, 40 mM NaH_2_PO_4_, pH 7.4, *MW*—molecular weight marker.

In hen’s egg yolk, protein fractions belonging to both globular glycoproteins and phosphitin complexes of the high-density lipoprotein were detected in gel after SDS-PAGE electrophoresis (Figure 4), including two egg-yolk allergens.

These fractions were identified based on molecular mass analysis and the available literature [2]. Additionally, an unrecognized fraction was once again found in samples from hens fed with soybean and lupine fodder. This fraction had a molecular weight of ~13 kDa (blue arrowhead). In the yolk, a protein with this molecular weight it is not known. The protein is present in a control sample, with a significant lower concentration. In the white, a common protein with a similar molecular weight is lysozyme (14.3) or cystatin (12.7 kDa) [20,21]. However, this protein (~13 kDa) was not detected in the corresponding samples of egg white proteins (i.e., in “S” and “L” egg-white samples); thus, the presence of this protein should not be a contamination of yolk extract by white proteins.

**Figure 5:**
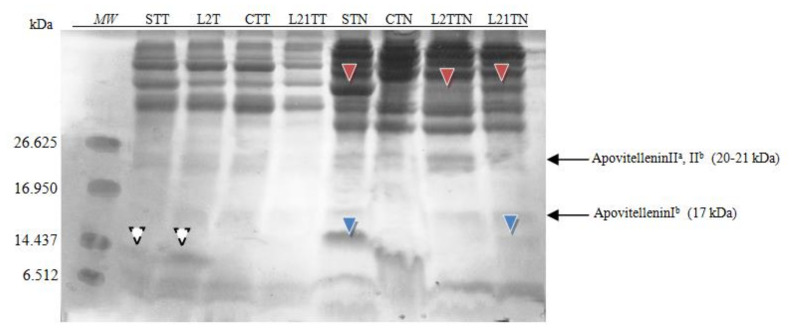
SDS-PAGE separation peptide profiles with Coomassie staining for egg yolk. Hen eggs were obtained after feeding the hens with soy, lupine or without adding legumes: S—soy for 21 days; C—control without legumes; L2—lupine for 2 days; L21—lupine for 21 days; TT—1% Tween 20 +0.4% Triton x-100; TN—1% Tween 20 + 0.4% Triton X-100, 280 mM NaCl, 40 mM NaH_2_PO_4_, pH 7.4, *MW*—molecular weight marker.

Similar to the egg-white extracts, the samples were subjected to peptide SDS-PAGE separation (in 16.5% gel). Considering the range of the size marker (6–26 kDa), two peptide fractions were identified (Figure 5). The main difference in the obtained peptide profile resulted from the extractant used.

After application of the TT extractant, in the STT and L2TT extracts, a fraction of ~13 kDa was identified (white arrowhead), which was also identified in the polyacrylamide gel. 

More protein and peptide fractions were identified after using TN as an extractant to yolk samples. Two additional fractions were present in the yolk extracts from eggs laid by hens fed with soybean-supplemented fodder. The presence of peptide ~15 kDa (blue arrowhead) was confirmed in the samples STN and LTN21. Moreover, an additional fraction ~37 kDa was detected (red arrowhead) in samples STN, L2TN and L21TN. The concentration of this fraction depends on the lupine feeding time (2 or 21 days), and for the STN sample, it comprises 5% of total protein. This fraction’s molecular weight is close to that of β-livetin (33–36 kDa). 

#### 2.2.3. Western-Blot Method

Protein and peptide fractions separated by SDS-PAGE electrophoresis were transferred by semi-dry electrotransfer on the PVDF membrane for Western blot analysis. The analysis was performed separately for the egg-white and egg-yolk samples. Eight different sera of children diagnosed with an allergy to different egg proteins, as well as to soy proteins, were used in this case (Table 2).

Images of membranes obtained after the Western-blot method with the application of sera from four patients diagnosed with egg protein allergy are presented.

#### 2.2.4. Egg White Protein Detection by Sera of Children Allergic to Eggs

**Figure 6:**
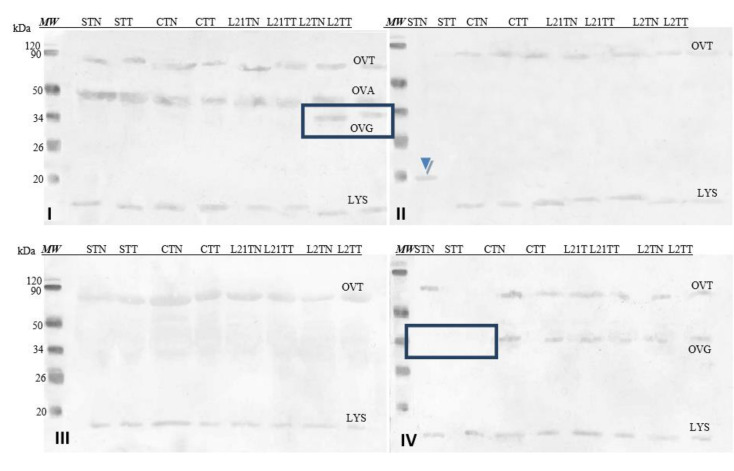
Images of the membrane obtained as a result of the Western-blot analysis with the sera of four patients allergic to eggs (**I**–**IV**) for egg’s white extracts. OVT—ovotransferrin, OVA—ovalbumin, OVG—ovoglobulin, LYS—lysozyme. Hen eggs were obtained after feeding the hens with soy, lupine or without adding legumes: S—soy for 21 days; C—control without legumes; L2—lupine for 2 days; L21—lupine for 21 days; TT—1% Tween 20 + 0.4% Triton x-100; TN—1% Tween 20 + 0.4% Triton X-100, 280 mM NaCl, 40 mM NaH_2_PO4, pH 7.4, *MW*—molecular weight marker.

In the case of egg white (Figure 6), the antibodies contained in the sera recognized some of the major fractions of the egg white, depending on the serum used. Antibodies present in serum No. I detected Gal d 3 (OVT), Gal d 2 (OVA) and Gal d 3 (LYS). In serum No. II, we found Gal d 3 and Gal d 4. Moreover, an unknown fraction with a molecular weight of approximately 20 kDa was identified as well (extracted by TN from S sample) (blue arrowhead). This fraction was also detected in that sample by SDS-PAGE electrophoresis (compare Figure 4). This could potentially indicate that this unidentified protein found in the egg white has significant immunoreactivity. The antibodies in serum No. III react with the protein fraction with a molecular weight of approximately Gal d 3 and the fraction of molecular weight by Gal d 4. Three protein fractions were identified by serum No. IV: Gal d 3, OVG and Gal d 4. There were differences in terms of the detection of OVG observed after immunostaining with sera I and IV, as well as the lack of detection of Gal d 3 and Gal d 4 by serum II in the STN sample.

#### 2.2.5. Egg-Yolk Protein Detection by Sera of Children Allergic to Eggs

**Figure 7:**
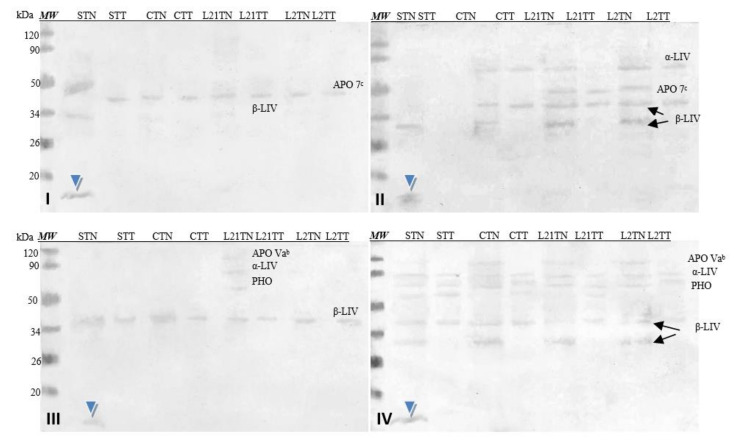
Images of the membrane obtained as a result ofWestern-blot analysis with the sera of four patients allergic to eggs (**I**–**IV**) for egg’s yolk. APO 7^b^—apovitellinin 7^b^, APO Va^b^—apovitellinin Va^b^, PHO—phosvitin, α-LIV—α-livetin, β-LIV—β-livetin. Hen eggs were obtained after feeding the hens with soy, lupine or without adding legumes: S—soy for 21 days; C—control without legumes; L2—lupine for 2 days; L21—lupine for 21 days; TT—1% Tween 20 +0.4% Triton x-100; TN—1% Tween 20 + 0.4% Triton X-100, 280 mM NaCl, 40 mM NaH_2_PO4, pH 7.4, *MW*—molecular weight marker.

Figure 7 shows images of membranes after Western-blot analysis of egg-yolk extracts by using four sera from egg-allergy patients. Serum antibodies detected various fractions present in the egg yolk, including yolk allergens (Gal d 6—YGP42 and Gal d 5—serum albumin of inactivating alpha-livetin). The antibodies present in each of the sera recognized an unidentified protein fraction with a molecular weight of ~13 kDa in the sample in which soy was a source of protein in the fodder (STN) (blue arrow).

#### 2.2.6. Egg-White Protein Detection by Sera of Children Allergic to Soy

Although no cross-reactivity has been reported in the literature for the antibody present in sera of patients allergic to chicken eggs and soy, due to the introduction of legumes into the fodder, an attempt was made to find differences in the immunoreactivity of obtained eggs with the application of sera of patients allergic to soy only. Four sera of patients diagnosed with a soy allergy and with no allergy to eggs were used.

**Figure 8:**
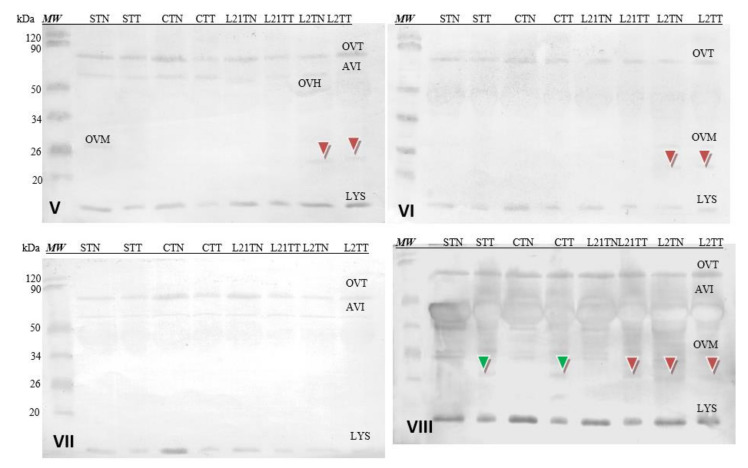
Images of the membrane obtained as a result of the Western-blot analysis with the sera of four patients allergic to soy (**V**–**VIII**) for egg’s white. OVT—ovotransferrin, OVA—ovalbumin, AVI—avidin, OVH—ovoinhibitor, OVM—ovomucoid, LYS—lysozyme. Hen eggs were obtained after feeding the hens with soy, lupine or without adding legumes: S—soy for 21 days; C—control without legumes; L2—lupine for 2 days; L21—lupine for 21 days; TT—1% Tween 20 + 0.4% Triton x-100; TN—1% Tween 20 + 0.4% Triton X-100, 280 mM NaCl, 40 mM NaH_2_PO_4_, pH 7.4, *MW*—molecular weight marker.

Soy-sensitized antibodies recognized all allergens of egg white, i.e., Gal d 3 (OVT) and Gal d 4 (LYS). Some of them (V, VII and VIII) also recognized AVI and Gal d 1 OVM in some samples (V, VI and VIII) (Figure 8). The differences in detection of test samples by these sera do not appear to be significant. Interestingly, the antibodies contained in sera V, VI and VII cross-recognized a protein fraction with a molecular weight of approximately 23 kDa (red arrowhead), which is not the major’s egg-white protein. This fraction was recognized in egg-white samples with the addition of lupine to chicken fodder (L21TT, L2TN and L2TT), but the reactivity was slight. However, serum VIII detected the protein also in sample STT and CTT.

#### 2.2.7. Egg Yolk Protein Detection by Sera of Children Allergic to Soy

**Figure 9:**
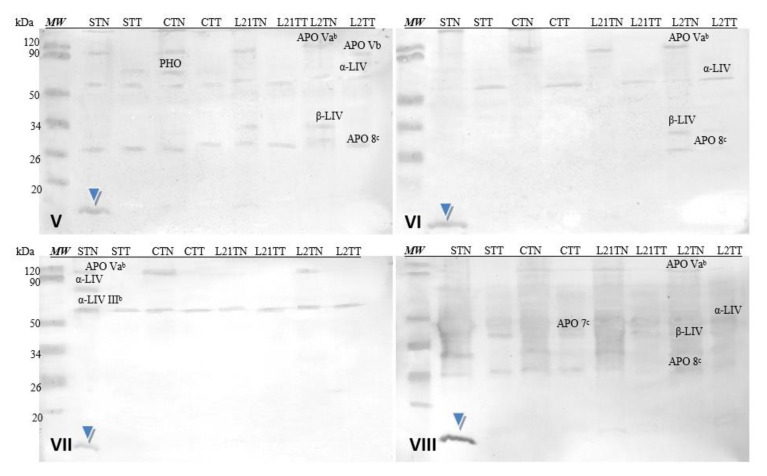
Images of the membrane obtained as a result of the Western-blot analysis with the sera of four patients allergic to soy (**V**–**VIII**) for egg’s yolk. APO Va^b^—apovitellinin Va^b^, APO Vb—apovitellinin Vb, PHO—phosvitin, APO 7^c^—apovitellin 7^c^, APO 8^c^—apovitellin 8^c^, α-LIV—α-livetin, β-LIV—β-livetin. Hen eggs were obtained after feeding the hens with soy, lupine or without adding legumes: S—soy for 21 days; C—control without legumes; L2—lupine for 2 days; L21—lupine for 21 days; TT—1% Tween 20 +0.4% Triton x-100; TN—1% Tween 20 + 0.4% Triton X-100, 280 mM NaCl, 40 mM NaH_2_PO4, pH 7.4, *MW*—molecular weight marker.

Corresponding analyses were also performed for hen’s egg-yolk proteins (Figure 9). The used sera did not detect yolk allergens (Gal d 5—α-LIV and Gal d 6—β-LIV) in all samples, because these allergens are not common for children [8]. However, antibodies contained in each of the used sera cross-recognized a protein fraction with a molecular weight of approximately 13 kDa (blue arrowhead). This fraction was present only in the STN sample (hens fed with soybean-supplemented fodder for 21 days). The identical results were demonstrated after the application of the sera of patients allergic to eggs (compare Figure 7). Additionally, the reactivity of this protein is really strong compared to others present in the prepared extracts (stronger by approximately 15%).

## 3. Discussion

Egg chemical composition, i.e., the yolk’s water level, lipid content and the average amount of protein, depends on the hen breed [22,23]. Based on the possibility of modifying the composition of eggs (fatty acids, fat-soluble vitamins, micronutrients, etc.), it can be expected that, by changing the composition of the fodder, differences in protein expression will also be visible. Few studies have investigated the impact of hen nutritional changes on the egg-protein profile. Current research on the effects of various protein sources contained in fodder on the protein composition of chicken eggs is limited and virtually does not touch on the problems of egg protein immunoreactivity. Instead, it concerns the possibility of increasing the content of proteins in eggs by using proven a biological activity, such as lysozyme or cystatin [20,21]. Kowalska (2019) indicates that the addition of narrow-leaved lupine to fodders instead of soybean meal results in reduced egg weight, improved yolk color (due to the increased amount of beta-carotene and lutein in the fodder) and increased protein quality measured in Haugh units [24].

In 2020, Troomer et al. [25] analyzed the possibility of protein transfer from fodder to chicken eggs and poultry meat. Using SDS-PAGE analysis and immunodetection with commercial anti-soybean and anti-peanut antibodies (Sigma-Aldrich, St. Louis, MO, USA; Lifespan Biosciences, Seattle, WA, USA), the transfer of soybean or peanut proteins into eggs and meat from fodder, as well as the resulting occurrence of allergenic soybean or peanut fractions in eggs, was found to be impossible [25].

It is well-known that many allergenic proteins are protease-resistant, which increases the likelihood of intact peptides up to seven amino acids long reaching the intestine and bloodstream [25]. Thus, the same phenomenon can be expected if hen fodder composition is modified.

### 3.1. Extraction

The composition of the extractant for further analysis, as well as its selection, is crucial in allergen analysis [12]. The literature describes various ways of extracting proteins from chicken eggs. The differences are primarily due to the type of extractant used, but also the extraction time and the ratio between the sample weight and the extractant volume. In 2011, Steinhoff compared 11 different ways of extracting proteins from both milk and egg white and egg yolk [18]. In this study, 6 extractants selected based on literature data were used [13,14,15,16,17,18]. According to Steinhoff, TN and 4% SDS are the best extractants for egg [18]. It can be expected that the beneficial impact of detergents used in his study on the efficiency of the protein extraction process was due to the presence of lipoproteins (especially in the yolk) in the samples. In the tests presented in this manuscript, the highest protein concentrations occurred in extracts obtained using 4% SDS, particularly in the case of egg white extracts. SDS used in such a high concentration interfered with the performed electrophoresis (due to its anionic nature), and since further analyses were based on immunochemical methods (Western blot) preceded by electrophoresis (SDS-PAGE), its quality was of paramount importance.

Hence, the choice of two extractants for further analysis, i.e., TN and TT, was determined not by the protein concentration in the samples (Table 1), but primarily by the quality of the electrophoresis and the highest number of protein fractions observed on the polyacrylamide gels (8 and 9 fractions in the case of egg white and 12–15 in the case of egg yolk). The extractants used enabled seven allergenic (it means, all known) protein fractions of chicken egg white and yolk to be extracted and observed on gels (Figure 1). Furthermore, the two solvents proved to be universal extractants for both egg-yolk and egg-white proteins. In contrast, other extraction methods recommended in the literature did not enable the detection of as many allergenic fractions.

### 3.2. SDS-PAGE Electrophoresis

Research indicates that changing the source of protein in fodder does not affect protein concentration but rather protein composition [19,26]. Extracts from the tested eggs were subjected to electrophoresis, using the Laemmli (1970) method [27], in a 14% gel. All known egg allergens in egg white and yolk were successfully identified. Apart from the major egg proteins (Gal d 1, Gal d 2, Gal d 3 and Gal d 4), differences in protein profile were also found between samples C, S, L2 and L21, while testing the chicken egg white by using the 14% gel. Two sample-differentiating protein fractions with molecular weights not corresponding to any common white protein were identified (Figure 2) [19]. The first, with a molecular mass of ~20 kDa (blue arrowhead), was found only in chicken egg white samples (STT and STN) obtained from eggs laid by hens fed with soybean-supplemented fodder. This indicates that soy proteins in the fodder may potentially affect the expression of new proteins in the egg. It may be an egg protein but with an altered primary structure, depending on the essential amino acids composition in the protein source (soy). This was also the case for samples of egg white from eggs laid by lupine-fed hens (L21TT and L2TT). They included a protein fraction with amass of ca. 23 kDa (Figure 2, red arrowhead). Compared to the control eggs, a decrease in the content of proteins with a molecular weight of ~17 kDa was observed as well, especially in eggs obtained from hens fed with soybean-supplemented fodder (STT and STN). At this stage of the study, it is impossible to indicate the mechanism responsible for the formation of the different proteins present in the samples. The origin of these new proteins should be carefully examined by proteomic analysis of fodder, eggs and blood. A lack of differences between the samples after peptide separation (in the 16.5% gel) may result from the gel’s structure and blocking protein separation (Figure 3).

Main yolk proteins and two major egg-yolk allergens (Gal d 5 and Gal d 6) were identified in chicken egg yolk by SDS-PAGE analysis in a 14% gel. An additional fraction (STN—ca. 13 kDa) was detected in egg samples in which soy was the source of protein in the fodder (Figure 4). In terms of molecular weight, this protein corresponds to cystatin (~12.7 kDa), which is a typical egg-white polypeptide that can also be found in the yolk in trace amounts [20]. However, it was not observed in the egg-white samples.

Differences in the protein composition of egg yolk were also found in the case of separation on peptide gel (16.5%). Using the TN extractant, significant differences were found in yolk samples depending on the fodders used. Compared to the control samples, the soybean-supplemented samples contain a significantly higher concentration of a fraction ~37 kDa, suggesting that soybean addition may have increased the β-livetin content in the yolk. The amount of this protein was also increasing during the period in which the hens received lupine-supplemented fodder and was significantly higher in samples obtained from hens that consumed such fodder for 21 days. Furthermore, the soybean sample includes a fraction of ca. 13 kDa (Figure 5), which cannot be clearly identified at this stage.

### 3.3. Western-Blot Method

To investigate differences in the immunoreactivity of the retrieved eggs, sera from patients diagnosed with an allergy to egg white only (4 sera) and soybean only (4 sera) were used.

In the case of extracts obtained from egg whites, the antibodies contained in the sera (1–4) recognized such major egg-white allergens as OVT (Gal d 3), OVA (Gal d 2) and LYS (Gal d 4), which served as a confirmation of the patients’ allergies. Additionally, in the case of serum II and the STN sample, the antibodies recognized a fraction with amass of ca. 20 kDa. This fraction was also identified during the SDS-PAGE electrophoresis. Unfortunately, we are unable to determine the origin of this fraction based on the available literature and preliminary studies conducted. It is only present in the soybean-supplemented fodder sample, based on which one may suggest that it occurs as a consequence of feeding soybean to hens. However, the weak intensity of this response and the fact that it occurred only with serum from a single patient does not suggest its clinical relevance (Figure 6).

The same sera were used to test the immunoreactivity of the obtained chicken egg-yolk samples. Their antibodies recognized the Gal d 6 and Gal d 5 allergens typical for egg yolk. Additionally, antibodies from all four sera recognized a protein fraction with amass of ca. 13 kDa, extracted with the TN solvent only from the yolk of eggs laid by hens fed with soybean-supplemented fodder—STN (Figure 7, blue arrow). Unfortunately, it is impossible to determine what protein this is at this stage of the study.

Due to the addition of legumes to the fodder, an attempt was made to find differences in the immunoreactivity of the obtained eggs by following the use of sera of patients allergic only to soybeans. Four sera of patients diagnosed with a soy allergy and no egg allergy were used.

While the antibodies present in these sera cross-recognized the basic protein fractions found in egg white and egg yolk, this response was very weak compared to the intensity of the reaction found in sera of subjects allergic to eggs.

In egg-white extracts, the antibodies recognized the major egg-white allergens. An antibody reaction with a protein fraction of ca. 23 kDa occurred as well in some samples (among them L21TT, L2TN and L2TT) (Figure 8).

Cross-reactivity of the sera with yolk protein was weaker. Similar to the sera of patients with egg allergy, the antibodies recognized a fraction of ca. 13 kDa in the egg yolk. This fraction was identified only in the STN sample. Moreover, this protein’s reactivity is really strong compared to others present in the prepared extracts (Figure 9, blue arrow) and nearly 10% stronger for serum 8 (grade 6). This suggests that anew fraction with potential allergenicity—particularly for soy-allergic individuals—appears in the egg yolk as a consequence of foddering hens with soy-based fodder, though it must be noted that the antibodies present in each of the sera used in the study (1–8) reacted with this fraction as well. Given its absence in the egg white, one might suspect that it is not any of the main milk proteins of similar molecular weight (neither cystatin nor lysozyme). This may be a protein with an altered amino acid sequence (primary structure) that appears in egg yolk when hens consume fodder containing soy. Considering the strength of this protein’s reaction with anti-soybean antibodies, its proteomic identification is worth considering.

Although Toomer et al. (2021) [25] found that eggs obtained from hens fed soybean- and peanut-supplemented fodder were free of soybean and peanut allergens, this study suggests that hen nutrition does affect egg allergenicity. The study by Toomer et al. (2021) [25] did not use patient sera but commercial antibodies directed against the respective allergenic proteins (peanut and soy), which significantly reduces the value of the results obtained. Based on the studies presented in this manuscript, new and unusual fractions exhibiting different antigenicity were found in eggs.

## 4. Materials and Methods

### 4.1. Material

#### 4.1.1. Sera

Four sera obtained from patients diagnosed with allergies to egg protein and four sera from patients with soy allergy were used in the study. The sera were obtained from the “SNOZ Alergologia Plus” Diagnostic and Treatment Centre for Allergology in Poznań, Poland. Application of sera obtained from the patients suffering from food allergies was approved by the Bioethical Commission of the Medical University of Karol Marcinkowski in Poznań, Poland (contract No. 671/17, 2017, and Annex 516/19). No sensitive data of the individuals involved were used in the experiment.

#### 4.1.2. Hen Eggs

The tested material was eggs provided by a local farm (chicken breed: Green-Legged Partridge). The hens were fed with fodder with or without the addition of legumes. The ground lupine and soy seeds were made available to the farm owner and incorporated into the fodder in the amount of 11% next to vegetables and fruits.

The tested material consisted of 40 eggs: 10 eggs obtained from hens fed with fodder without the addition of legumes (control samples—C), 10 eggs obtained from hens fed with soybeans-supplemented fodder for 21 days (S), 10 eggs obtained from hens fed with lupine-supplemented fodder for 2 days (L2) and 10 eggs from hens fed with lupine-supplemented fodder for 21 days (L21). Fresh eggs from individual groups were broken and separated into yolks and egg whites, and then pooled and frozen for further analysis.

### 4.2. Methods

#### 4.2.1. Extraction

In order to extract the protein from eggs, appropriate buffers were used, which are declared for the extraction of allergenic proteins from eggs and legumes. The extraction quality was compared for6 extractants recommended in the literature: (1) 1% Tween 20 + 0.4% Triton x-100 (1:10 *v*/*v*) [17]; (2) 4% SDS (1:20 *v*/*v*) [16,18]; (3) acidified with 1M HCl dH_2_O, pH 6, (1:3 *v*/*v*) [15]; (4) phosphate-buffered saline PBS (1:10 *w*/*v*) [13]; (5) PBST pH 7.4, i.e., PBS buffer with 0.05% of Tween 20 (1:10 *v*/*v*) [13]; and (6) 1% Tween 20 + 0.4% Triton X-100, 280 mM NaCl, 40 mM NaH_2_PO_4_, pH 7.4 (1:10 *v*/*v*) [18].

#### 4.2.2. Protein-Content Determination

The concentration of protein in the prepared extract was determined with the Smith method (1985) to compare extraction efficiency [28].

#### 4.2.3. SDS-PAGE Electrophoresis

The resulting extracts (applied in a volume of 1.5 µL) were separated by electrophoresis on a 14% polyacrylamide gel under denaturing conditions [27] and gradient gels 16.5% MP Tris-Tricine (4563064, Bio-Rad, Hercules, CA, USA) in a volume of 5 µL. The following were used as the mass molecular marker: Polypeptide SDS-PAGE Marker (Bio-Rad 161-0326, Hercules, CA, USA) with the range of 1.4–26.6 kDa and a Prestained Protein Molecular Weight Marker (Thermo Fisher Scientific, 26612, Waltham, MA, USA) with the range of 14–120 kDa. The protein pattern was visualized by silver staining and for peptide gel Coomassie Brilant Blue staining. The gels were documented by using CLIQS (TotalLab Quant, Newcastle-Upon-Tyne, UK).

#### 4.2.4. Western-Blot Method

Protein and peptide fractions separated by SDS-PAGE electrophoresis were also transferred by a semi-dry electrotransfer to a polyvinylidene difluoride membrane with a special porosity of 0.2 and 0.45 μm (Immobilon-P, Merck Millipore Ltd., Burlington, MA, USA). The current was 200 mA for 30 min and then 120 mA for 90 min.

Sera from outpatient with hen-egg allergies were diluted in 1% BSA solution in TBS–Tween (1:20 *v*/*v*). Goat anti-human IgE polyclonal antibodies labeled with alkaline phosphatase diluted 1:2500 in TBS-T (A18802, Invitrogen, Waltham, MA, USA) was used as Ab II antibodies. The membranes were analyzed using the CLIQS program (TotalLab Quant, Newcastle-Upon-Tyne, UK).

## 5. Conclusions

Preliminary studies conducted on the effect of fodder composition on chicken-egg protein profile suggest that the addition of soybeans and lupines to the fodder modifies egg-protein expression. The identified protein fractions differing from those found in the controls are likely to be egg proteins whose amino acid composition was slightly modified as a consequence of providing hens with a different protein source in the fodder (essential amino acid supply) or transfer of short peptides from the fodder; they may also be proteins that broke down into other polypeptide fractions as a consequence of the modification. Further analyses using MS are necessary to accurately identify these protein fractions and provide definite answers.

The differences found in the immunoreactivity of the proteins contained in the chicken egg-white samples do not appear to be as significant as the occurrence of a protein with a molecular weight of ~13 kDa in the yolk of eggs laid by soybean-fed hens. This protein reacts both with four sera of children allergic only to eggs and those four allergic only to soy. Since this response is very strong, it is worth confirming its clinical relevance. The results are worthy of future studies, because of cross-reactivity sera children allergic solely to that fraction. Thus, this protein (~13 kDa) presence in yolks may increase immunoreactivity of eggs for children allergic solely to soy.

## Figures and Tables

**Table 1:** Protein concentration depending on the extractant used.

Sample	Protein Content (mg/g)
PBS white	74.51 ± 2.88
PBS yolk	60.02 ± 0.95
PBST white	89.80 ± 1.15
PBST yolk	59.68 ± 2.98
H_2_O white	44.86 ± 1.49
H_2_O yolk	31.73 ± 0.33
SDS white	196.55 ± 19.81
SDS yolk	77.51 ± 4.02
TT white	65.19 ± 1.27
TT yolk	34.65 ± 3.32
TN white	51.42 ± 0.96
TN yolk	68.74 ± 0.82

**Table 2:** Presentation of used sera (A and B).

(A) Sera of Children Allergic to Egg						
**Sera**	**Egg Allergy**					
	**Protein**		**Yolk**		**Whole Egg**	
	**Class**	**IgE Content** **(kU/L)**	**Class**	**IgE Content** **(kU/L)**	**Class**	**IgE Content** **(kU/L)**
I	6	>100	2	0.7–3.5	-	-
II	-	-	-	-	2	0.7–3.5
III	1		2	0.7–3.5	2	0.7–3.5
IV	3	3.5–17.5	-	-	1	<0.7
**(B) Sera of Children Allergic to Soy**						
**Sera**	**Soy Allergy**					
	**Class**			**IgE Content** **(kU/L)**		
V	4			~50		
VI	6			>100		
VII	3			3.5–17.5		
VIII	6			>100		

## Data Availability

The data presented in this study are available on request from the corresponding author.

## Sample Availability

Samples of the compounds are not available from the authors.

## List of Abbreviations

TT	1% Tween 20 + 0.4% Triton x-100
TN	1% Tween 20 + 0.4% Triton X-100, 280 mM NaCl, 40 mM NaH_2_PO_4_, pH 7.4
S	addition soy to fodder
C	control/fodder without legumes
L2	addition lupine to fodder for 2 days
L21	addition lupine to fodder for 21 days
OVT	ovotransferrin
OVA	ovalbumin
OVG	ovoglobulin
OVM	ovomucoid
LYS	lysozyme
β-LIV	β-livetin
APO 7^c^	apovitellin 7^c^
α-LIV	α-livetin
APO Va^b^	apovitellinin Va^b^
PHO	phosvitin
OVH	ovoinhibitor
AVI	avidin
APO Vb	apovitellinin Vb
APO 8^c^	apovitellin 8^c^
α-LIV III^b^	α-livetin III^b^
